# Lineage-associated differences in adenine methylation patterns of mammalian-associated *Campylobacter fetus* isolates: a possible role for epigenetic factors in host tropism and pathogenesis

**DOI:** 10.1128/spectrum.00954-26

**Published:** 2026-06-30

**Authors:** Susan Nadin-Davis, Marc-Olivier Duceppe, Mingsong Kang, Emily Hoover, John Devenish

**Affiliations:** 1 Ottawa Laboratory Fallowfield, Canadian Food Inspection Agency 5737, Ottawa, Ontario, Canada; Michigan State University, East Lansing, Michigan, USA

**Keywords:** epigenetics, adenine methylation motifs, phylogeny, methylase, genomics

## Abstract

Mammalian *Campylobacter fetus* (CF) is divided into two subspecies, *C. fetus fetus* (CFF) and *C. fetus venerealis* (CFV), the latter being bovine-adapted and responsible for the notifiable disease bovine genital campylobacteriosis (BGC). Differentiation between CF subspecies has traditionally been undertaken by a few biochemical tests, but these are complicated by the existence of a biotype, *C. fetus venerealis* intermedius (CFVi), which shares attributes of both CFF and CFV. Molecular methods targeting specific genes have gained acceptance for more accurate subtype identification and align well with whole-genome analysis. However, limited genomic diversity between subtypes has confounded efforts to understand the genetic basis for differential host tropism and pathogenesis of these organisms. A previous study of a small cohort of *C. fetus* isolates suggested that *dam* gene coding variations might correlate with CF subtype. Accordingly, this study examines a cohort of 331 *C. fetus* genomes, representative of all seven phylogenetic groups for their complement of adenine methylases and the genomic motifs they target in representative isolates. All CF isolates retained a *cfeM1* gene, the presence of which correlates with RAATTY methylation, while seven other adenine methylase genes exhibited distinct cladal distributions. Notably, a *cjeM1* gene appears to target the CCAN7TAG/CTAN7TGG motif in CFV and CFVi isolates only. Given the increasing recognition of the impact of adenine methylation on bacterial-host interactions, further exploration of the role of adenine methylation in *C. fetus* pathogenesis could reveal mechanisms contributing to BGC and thus aid in its eradication.

**IMPORTANCE:**

*Campylobacter fetus* remains an important zoonotic pathogen, for which a better understanding of its host tropism and pathogenesis is sought. However, the limited genomic variation observed between subtypes has to date confounded efforts in this regard. This study suggests that an alternative approach that examines epigenetic differences between subtypes, specifically adenine methylation patterns, may reveal mechanisms critical to the pathologies of these organisms.

## INTRODUCTION

Of the three currently recognized subspecies of *Campylobacter fetus*, the genetically most divergent is *C. fetus* subsp. *testudinum*, which is usually associated with reptilian hosts (1, 2), while the other two, *C. fetus* subsp. *fetus* (CFF) and *C. fetus* subsp. *venerealis* (CFV), are responsible for mammalian infections, principally in ruminants (3). These latter two subspecies exhibit distinct host tropisms and disease pathologies (4). Following fecal-oral spread from environmental sources, CFF resides in the gastrointestinal tract of ruminants, from which a descending systemic infection to the reproductive tract in pregnant cows and ewes leads to early embryonic death and abortion. Similarly, CFF can also cause serious dead-end host septicemic infections in elderly and immunocompromised humans (5). In contrast, CFV is found only in the reproductive tract of cattle and is believed to cause an ascending non-systemic infection in cows, resulting in infertility and abortion. This pathogenesis is considered important to the bovine industry and defines the notifiable disease of bovine genital campylobacteriosis (BGC) (6). Disease classification of CFV is complicated by the presence of a specific biovar, *C. fetus* subsp. *venerealis* biovar intermedius (CFVi), which has been isolated from both the intestinal and reproductive tracts of cattle. The etiological role of CFVi in BGC has never been conclusively determined, but it is currently aligned with that of CFV in this regard. The high prevalence of BGC in some countries results in significant economic losses to the cattle industry, thereby motivating control efforts (7, 8). Given this difference in disease priority and notification between the subspecies, timely and accurate differentiation of CFV and CFVi from CFF strains is needed.

Traditionally, *C. fetus* subspecies differentiation has involved the application of a limited number of specific biochemical tests (6). These include evaluation of growth on media containing 1% glycine, generation of H_2_S from cysteine-rich media, selenite reduction capability, and growth at 42.5°C. CFF is usually positive in all four tests, and CFV is negative in each test, while CFVi isolates exhibit an intermediate phenotype, showing positive for the generation of H_2_S and selenite reduction, like CFF, but negative for growth on media at 42.5°C or containing 1% glycine, like CFV (9). Unfortunately, these phenotypic tests have been reported as unreliable for identification purposes (10). They are labor-intensive with long incubation periods, can be challenging to interpret, and suffer from repeatability problems, which may be explained by a lack of standardization between laboratories. Accordingly, alternative genetic-based discriminatory methods have been developed. While increasing genome length and sequence rearrangements in the order CFF < CFV < CFVi have been reported (11), consistent with the ability of pulse-field gel electrophoresis and amplified fragment length polymorphism to discriminate between CFF and CFV (12, 13), these procedures are technically challenging for diagnostic purposes. PCR-based detections of two markers, a CF-specific *nahE* gene and a CFV-specific insertion sequence ISC*fe1* (14–16), are reported to accurately discriminate between CFF and CFV (17). Further classification relies on the genetic basis for phenotypic differences in the ability to metabolize cysteine to generate H_2_S; in both CFF and CFVi, this correlates with the presence of a complete L-cysteine transporter operon comprising three genes (*tcyA*, *B*, and *C*) (18). CFV isolates lacking this ability have a deletion in which the 3′ terminus of the *tcyA* gene is spliced to a portion of the *tcyC* gene, thereby eliminating the *tcyB* open reading frame (ORF); a PCR targeting this genetic difference accurately predicts this metabolic distinction (19) and provides a means of discriminating between CFV and CFVi isolates. Other approaches for subspecies discrimination have proposed targeting specific single nucleotide polymorphisms (SNPs) (20).

Despite these genetic tools for subtype discrimination, the underlying genomic differences responsible for the distinct pathology caused by these pathogens remain unknown. Studies of the genomic organization of *C. fetus* isolates have revealed the clonal nature of this species and its high level of synteny, with differences limited to small regions that often contain pathogenicity factors but do not entirely correlate with subspecies (2, 18, 21, 22). It has been well documented that *C. fetus* isolates normally segregate into two serotypes, A and B, reflecting differences in the *sap* genes that encode surface layer proteins (slps). All serotype B isolates are considered CFF, while serotype A isolates can be of either subspecies, and some isolates are typed as AB (23, 24). While these slps are considered important virulence factors (25), their lack of segregation between subspecies precludes a primary role in eliciting distinct subspecies pathology.

Several phylogenetic studies employing whole-genome sequence data divide the mammalian-associated CF strains into seven clades, of which two represent serotype B CFF isolates, while the remainder comprise serotype A (26–29). Isolates of one recently emerged serotype A clade (clade 1) are strongly bovine-associated, are generally phenotypically representative of CFV and CFVi (26, 27, 30), and retain one or more copies of the ISC*fe1* marker. Variation in the sequence of this locus, however, appeared to correlate with further subdivision of clade 1 into two subclades, 1a and 1b (26). Additional SNPs present exclusively in clade 1 are potential alternative markers of this subspecies (26). Three genes of the accessory genome, *mat1*, *glf,* and *wcbK*, are differentially retained by CF clades (22), though the roles these genes might play in host-cell interactions remain unclear. Other studies suggest that, compared to CFF, CFV and CFVi retain a greater number of genes involved in quorum sensing and membrane transport. However, it was also acknowledged that many other differences were due to genes of unknown function (30). Multi-locus sequence typing appears to exhibit some congruence with the cladal structure of CF phylogeny (26), but this method was deemed to have limited value as a CF subtyping tool (29).

DNA methylation occurs widely in prokaryotes, often as part of a restriction-modification (R-M) system, which protects the bacterial genome from invasion by unmethylated foreign DNA (31). Some “orphan” DNA methyltransferases (MTases) function independently of these restriction mechanisms, and in recent years, DNA modification, including methylation, has been increasingly recognized as an important means of gene regulation (32, 33). The most common base methylation in bacteria involves transfer of a methyl group from the substrate *S*-adenosyl-L-methionine to the exocyclic N6 nitrogen of an adenine residue contained in a specific sequence motif. Extensive studies on the genome methylation of gamma-proteobacteria by the *dam*-encoded DNA adenine methyltransferase have demonstrated its role in controlling the expression of many genes (34, 35) through various mechanisms (36), thereby providing an important additional level of regulatory complexity beyond that conferred by DNA base sequence alone (36). The importance of this epigenetic process to host-pathogen interactions was illustrated in *Salmonella typhimurium*, for which loss of *dam* gene function strongly attenuates virulence (37). Furthermore, phase variation in adenine methylation can contribute to the phenotypic heterogeneity of many pathogens and facilitate host colonization, immune evasion, and virulence (33, 34, 36).

A few studies have explored the role of methylation in members of the *Campylobacter* genus. *Campylobacter jejuni* encodes multiple R-M system methylases (38), and variable methylation patterns have been linked to differences in gene expression and virulence (39, 40). One report described a gene encoding an orphan adenine MTase important for cell motility and host cell invasion (41). Methylation at GATC sites has been detected in isolates of some *Campylobacter* species (42). A methylome study of *Campylobacter coli* identified eight distinct adenine methylation motifs (43), and host-dependent adenine methylation at the GATC motif has been described in this species (44). To date, we are unaware of similar studies in *C. fetus;* however, annotation of 14 polished CF genomes identified various *dam* gene homologs that were distributed differently between phenotypes (11).

Given the increasing recognition of the role of epigenetic modification in modulating host-pathogen interactions, variation in adenine methylation between CF subspecies was explored. An initial study examined whether phenotypic characterization of 33 *C. fetus* isolates using highly standardized procedures could differentiate CF subspecies and biovar type accurately in comparison with whole-genome sequencing (WGS). The phylogenetic placement of these isolates, together with 298 additional *C. fetus* genomes, clearly identified seven clades, including one bovine-associated clade representative of CFV/CFVi subtypes. Screening of this sequence collection for genes encoding adenine MTases revealed variations strongly correlated with the species phylogeny, a feature reinforced by interrogation of the methylome of representative isolates. The potential role of DNA methylation in *C. fetus* pathology is discussed.

## MATERIALS AND METHODS

### Isolate growth and phenotypic characterization

Of the 31 *C. fetus* field isolates included in this study (Table 1), 13 originated from clinical specimens from bulls submitted for culture from artificial insemination facilities across Canada. These preputial wash samples were inoculated into Clark’s transport enrichment medium and then submitted to the Animal Health Microbiology Laboratory (AHML) for culture, as previously described (45). The remaining isolates had been collected from various sources around the world. Two additional isolates available from the American Type Culture Collection were also included for comparative purposes. Isolates were inoculated into vials of CryoStor cryopreservative solution (Innovatek Medical Inc., Vancouver, Canada) and frozen at −80°C for long-term storage as part of a stock culture collection maintained by the AHML. For analysis in this study, *C. fetus* isolates were thawed and cultured on Cysteine Heart blood agar or Mueller-Hinton agar containing 10% sheep blood under microaerophilic conditions (4% O_2_, 9.5% CO_2_, and 86.5% N_2_) for 2–4 days at 36°C. Pure culture isolates were identified to genus, species, and serotype (A or B) using a highly specific *C. fetus* monoclonal-based ELISA procedure (46). Subspecies and biovar identification were performed as previously described (11) by scoring each isolate for up to four phenotypes: growth at several concentrations of glycine with a definitive evaluation using a cutoff of 1.1% glycine, H_2_S production in L-cysteine-containing media, selenite reduction ability, and growth at 42.5°C. Only the first three phenotypes were employed for all isolates, as growth at 42.5°C was found to be inconsistent for some samples.

**TABLE 1:** Source and characterization of 33 *Campylobacter fetus* isolates by phenotype and phylogenetic analysis^*a*^

Isolate designation	Isolate source and original subtype identification					Phenotypic test results at AHML		Type by WGS	
	Country/region of origin	Year of isolation	Year received at AHML	Species/sample type	Original ID of isolate	c-ELISA serotype	AHML phenotypic ID	Genetic ID	Clade assignment
00AI-031^*b*^	Canada/British Columbia	2000	2000	Bovine/PW	CFF	A	CFF	CFF	2
02AI-725^*b*^	Canada/Alberta	2002	2002	Bovine/PW	CFF	A	CFF	CFF	7
06AI-1283	Canada/Ontario	2006	2006	Bovine/PW	CFF (B)	B	CFF	CFF	5
08AI-948^*b*^	Canada/Alberta-Premise 1/Animal 1	2008	2008	Bovine/PW	CFV	A	CFV	CFV	1a2
08AI-988	Canada/Alberta	2008	2008	Bovine/PW	CFF (B)	B	CFF	CFF	5
08AI-1038	Canada/Alberta-Premise 1/Animal 2	2008	2008	Bovine/PW	CFV	A	CFV	CFV	1a2
08AI-1102-33B	Canada/Alberta-Premise 2/Animal 1	2008	2008	Bovine/PW	CFV	A	CFV	CFV	1a2
08AI-1102-42A^*b*^	Canada/Alberta-Premise 2/Animal 2	2008	2008	Bovine/PW	CFV	A	CFV	CFV	1a2
09AI-980^*b*^	Canada/Quebec	2009	2009	Bovine/PW	CFF	A	CFF	CFF	2
10AI-182	Canada/Ontario	2010	2010	Bovine/PW	CFF (B)	B	CFF	CFF	5
19AI-311	Canada/Quebec	2019	2019	Bovine/PW	CFVi	A	CFVi	CFF^*e*^	7
ADRI-510	Canada/British Columbia	1960	ND	Unknown	CFV	A	CFVi^*d*^	CFVi	1b
ADRI-513	Australia	ND	ND	Unknown	CFVi	A	CFVi	CFVi	1b
ADRI-516	Unknown	ND	ND	Unknown	CFV	No result^*c*^	CFF^*d*^	CFF	2
ADRI-521	Jamaica	ND	ND	Bovine/unknown	CFV	A	CFV	CFV	1a2
ADRI-530	Canada/Quebec	ND	1982	Unknown	CFV	A	CFV	CFV	1a2
ADRI-544	Australia/Queensland	1984	ND	Bovine/vaginal mucus	CFVi	A	CFVi	CFVi	1b
ADRI-545^*b*^	Australia/Northern Territory	1984	ND	Bovine/reproductive tract	CFVi	A	CFVi	CFVi	1b
ADRI-547	Australia/South Australia	ND	ND	Bovine/bull prepuce	CFF	A	CFF	CFF	7
ADRI-555	US/Montana	ND	1985	Unknown	CFV	A	CFVi^*d*^	CFVi	1b
ADRI-804	England	ND	1985	Bovine/vaginal swab	CFV	A	CFVi^*d*^	CFVi	1a1
ADRI-838	US/Montana	ND	1986	Unknown	CFV	A	CFVi^*d*^	CFVi	1a1
ADRI-865	Belgium	ND	1986	Bovine/bull prepuce	CFV	A	CFV	CFV	1a2
ADRI-1025	Sweden	ND	ND	Human	CFF	A	CFF	CFF	6
ADRI-1031	Sweden	ND	ND	Unknown	CFV	A	CFV	CFV	1a2
ADRI-1335	Argentina	ND	ND	Unknown	CFF	A	CFVi^*d*^	CFVi	1a1
ADRI-1337	Argentina	ND	ND	Unknown	CFF	A	CFVi^*d*^	CFVi	1a1
ADRI-1359	Argentina	ND	ND	Unknown	CFV	No result^*c*^	CFF^*d*^	CFVi^*e*^	1a1
ADRI-1362^*b*^	Argentina	1989	ND	Unknown	CFV	A	CFVi^*d*^	CFVi	1a1
ADRI-1490	US/Tennessee	ND	ND	Unknown	CFF	A	CFF	CFF	2
ADRI-1554	Sweden	ND	ND	Unknown	CFF	A	CFV^*d*^	CFV	1a2
ATCC19438	England	1962	ND	Bovine/vaginal mucus	CFV	A	CFV Type Strain	CFV	1a2
ATCC27374	France	1952	ND	Ovine/fetal brain	CFF	B	CFF Type Strain	CFF	4

^*a*^ ND, no date recorded; PW, preputial wash.

^*b*^ Isolates sequenced in a previous study (11).

^*c*^ No result indicates isolate had a rough lipopolysaccharide phenotype.

^*d*^ Discrepancy between original isolate phenotype and phenotype determined at AHML.

^*e*^ Discrepancy between AHML phenotypic assignment and genomic typing.

### Illumina sequencing

Seven isolates in this study had been sequenced previously (11). For the remaining 26 isolates listed in Table 1, DNA was extracted from colonies collected from agar plates using a Wizard Genomic DNA extraction kit as per the supplier’s directions (Promega) and quantified by a Qubit instrument using a broad-sensitivity dsDNA quantification kit (Thermo Fisher Scientific). DNA dilutions were employed in the generation of indexed Nextera XT libraries that were run on a MiSeq instrument to generate 2 × 250 paired-end reads, except for CFF-19AI-311 (2 × 300 bp paired-end reads [MiSeq]) and CFV-08AI-1102-33B (2 × 100 bp paired-end reads on a HiSeq 2500 instrument).

### Nanopore sequencing

The Nanopore sequencing library was prepared from the same DNA sample using the Native Barcoding Kit SQK-NBD112.24 (Oxford Nanopore Technologies) without shearing and sequenced on a FLO-MIN112 flow cell using a MinION Mk1B device. Another library was prepared by first submitting the same DNA to whole-genome amplification (WGA) using the REPLI-g Mini Kit (Qiagen, USA), following the manufacturer’s recommendations. Nanopore reads were basecalled using Dorado v0.9.1 (https://github.com/nanoporetech/dorado) in super-accuracy mode.

### Short-read assembly

Samples for which only Short Read Archive (SRA) short paired-end reads were available were assembled with SKESA v2.5.1 (47) using the “--sra_run” flag. Samples for which only short paired-end reads were generated in-house were trimmed and filtered with fastp v0.23.4 (48) and assembled with SKESA v2.5.1 (47). See https://github.com/duceppemo/BGA_short for details.

### Hybrid assembly

Filtlong v0.2.1 (https://github.com/rrwick/Filtlong) was used to filter out the bottom 5% nanopore reads. The remaining long reads were assembled with Flye v2.9.3 (49). The assemblies were first polished with the same long reads using Medaka v1.11.3 (https://github.com/nanoporetech/medaka) and then polished again with trimmed short paired-end reads using a combination of Polypolish v0.6.0 and pypolca v0.3.1 (50) with the “careful” flag. The sequencing coverage depth was determined using Minimap2 v2.26 (51) and SAMtools v1.18 (52) for both Nanopore and Illumina reads. Default parameters of the bioinformatics software were used unless otherwise specified. See https://github.com/duceppemo/BGA for details.

### Phylogenetic analysis

A total of 314 CF genome sequences were downloaded from the NCBI. The quality of these sequences was examined using CheckM2 v1.1.0 (53), resulting in the exclusion of nine genomes (contamination >2% or specific completeness <95%). The remaining 305 genome assemblies, together with the 26 *C. fetus* strains sequenced and assembled in this study, were used for phylogenetic analysis (Table S1). When Illumina paired-end data were available from the Short Read Archive for published strains (*n* = 272), a SKESA v2.5.1 assembly was produced for inclusion in the analysis, reducing biases caused by comparing assemblies created with different assemblers. Downloaded assemblies were used (*n* = 33) when no short reads were publicly available for a specific strain.

Core SNPs between strains were detected using the alignment-based Parsnp v2.1.5 method (54), employing sequence GCF_011600945.2 (sample 00AI-031) as reference. Phylogeny was inferred using FastTree v2.2.0 (55) with the “gtr” model and 10,000 bootstraps.

### *In silico* PCR

BLAST-based *in silico* PCR was performed (https://github.com/duceppemo/insilicoPCR) using version 0.410, employing four sets of PCR primers/probes previously published for the targets *nahE*, ISC1, and ISC2 contained within the ISC*fe1* sequence and a *tcyB* deletion (Table S2).

### Genome marker screening

Alleles of each gene target were downloaded from representative *C. fetus* isolates and aligned in MEGA 7 (56). Well-conserved regions of relatively high GC content, usually 100 bases in length, were identified and used for gene screening (Table S3). Shorter sequences were employed to minimize sequence splitting between contigs. *C. fetus* genomes were initially screened for the *dnaA* and 16S *rRNA* genes as internal controls, *sapA* and *sapB* genes, which are clade-specific, and a transposase associated with IS605 transposons previously reported to be selectively retained within CFV/CFVi genomes (11). Since the original intention was to avoid any assembly bias, BLAST screening of SRA data was performed directly from the NCBI database wherever possible. The presence of more than 10 sequences with >80% match over >80% of the query sequence was considered highly indicative of the presence of the gene, and in most cases, the BLAST search retrieved the maximum number of 100 hits. Limitations in the accessibility of some SRA data, which had been aligned to the reference CFF (strain CFF-82-40), precluded the scoring of genes present in that isolate (i.e., 16S *rRNA, dnaA*, and *sapA*). These sequences were screened in the NCBI interface using assembled FASTA files. Such files were also used for additional screening of longer target sequences, where necessary, to better define specific, unusual profiles.

NCBI annotation of 14 representative CF genomes (11) and screening using the REBASE database identified eight variously retained genes responsible for adenine MTase activity (*cjeI, dam, EcoRIM, cfeMI, cfeMII, cfeMIII, cfeMIV*, and *hsdM*). For screening assembled FASTA files of a larger CF cohort for seven of these genes (*cjeI, EcoRIM, cfeMI, cfeMII, cfeMIII, cfeMIV*, and *hsdM*), the complete ORFs of selected isolates were used in BLAST searches to allow for the frequent identification of indels. Three *dam* genes of differing lengths due to variation within their 5′ termini were predicted (*dam1–3*). Scoring for these three alleles was performed using BLAST analysis with three distinct query sequences, each 50–60 bases in length and representative of the different 5′ terminal regions, along with the conserved 3′ terminal sequence. Additional screening was undertaken using an internal sequence to discriminate between the two *dam2* alleles. All sequences used for these screens are provided in Table S3.

### Genome methylation analysis

#### PacBio

Data from five isolates (CFF-02AI-725, CFF-09-AI-980, CFV-08AI-948, CFVi-ADRI-545, and CFVi-ADRI-1362), sequenced using PacBio RSII technology as previously described (11), were analyzed for evidence of methylation using the SMRT Link v7.0.1 software suite.

#### Nanopore

In addition, seven isolates (CFF-02AI-725, CFF-00AI-031, CFF-10AI-182, CFF- ATCC27374, CFV- 08AI-948, CFV- 08AI-1102, and CFVi-ADRI-545) for which both native and WGA DNA were sequenced using the MinION (Oxford Nanopore Technologies) were studied for evidence of methylation using nanodisco v1.0.3 (57). Nanopore reads were base-called with Guppy v6.3.8 with the “--fast5_out” option for nanodisco analyses.

### Motif distribution analysis

Whole genome sequences in FASTA format, together with their respective annotations in GFF format, were submitted to the online analysis tool DistAMo (available at https://www.computational.bio.uni-giessen.de/distamo/) for analysis of the distribution of certain sequence motifs identified by the methylation analysis (58). This tool employs algorithms to calculate the relative abundance of short motifs using codon redundancy and quantifies their under- or over-representation by a *z*-score.

All bioinformatic analyses were performed using default parameters unless stated otherwise.

## RESULTS

### Phenotypic analysis

Given the reported challenges in phenotypic classification of CF isolates, initial studies reassessed whether the application of standardized biochemical testing in a single laboratory could yield results congruent with those obtained from genomic analysis performed in parallel. A cohort of 33 *C. fetus* isolates, including 31 field isolates originating from a variety of sources, was phenotypically evaluated together with two type strains included for comparison (Table 1). According to established AHML protocols for glycine growth tolerance, all CFF strains grew at glycine concentrations >1.1%, whereas CFV and CFVi isolates were found to tolerate glycine concentrations up to 1.1% but never above. Regarding H_2_S production from cysteine and selenite reduction, all CFV strains were negative for both tests, whereas virtually all CFF and CFVi isolates were positive. Antigenic characterization of all 33 isolates identified 27 as serogroup A and 4 as serogroup B, while two yielded no result due to a rough lipopolysaccharide. The two type strains included in this group yielded phenotypes completely consistent with their prior identification.

Twenty-two of these isolates had previously been phenotypically typed by other laboratories. Phenotypic testing at AHML yielded consistent subspecies and biovar identifications for 23 isolates, while the remaining 10 gave inconsistent results between laboratories. The most common variation was in the discrimination between CFVi and CFV (*n* = 5), while differentiation between CFF and CFV was problematic for three isolates, and a further two isolates gave inconsistent identities between CFF and CFVi. Final identifications based upon genetic characterization undertaken at AHML are indicated in Table 1.

### Phylogenetic and *in silico* PCR analyses

**Fig 1:**
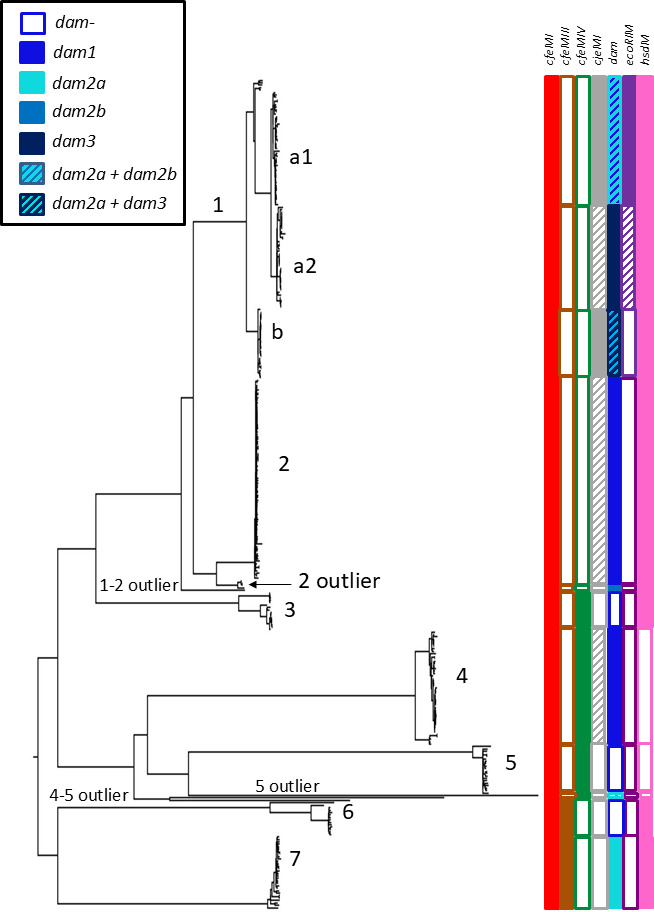
Phylogeny of 331 mammalian CF genomes based on their core genomes. Clades and subclades described in the text are identified at nodes or to the right of the cluster. At the far right, seven columns indicate the predominant genotype of seven DNA methyltransferases within each clade. Gene presence (colored fill) or absence (white fill) within each clade is indicated with a hatched pattern indicating a mixed genotype. The inset at top left identifies the color coding used to denote the various *dam* genotypes. Data for *cfeMII* are not given because so few isolates contained this gene.

Of the 33 phenotyped isolates, WGS was generated for 26 using Illumina technology; seven of these isolates had been sequenced previously (11). These sequences were combined with a database of 298 *C. fetus* sequences of mammalian origin retrieved from the NCBI (Table S1) and used to generate a phylogenetic tree (Fig. 1). Consistent with prior findings, the resulting tree identified seven main branches, denoted 1–7. As summarized in Table 2, *in silico* PCR analysis of this cohort clearly identified those in clades 2–7, as well as eight outlying samples, as CFF based on their ISC*fe1*−, *tcyB*+ genotype. Clade 1, which was highly bovine associated as expected, was subdivided into three subclades, of which subclades 1a1 and 1b represented CFVi isolates based on their ISC*fe1*+, *tcyB*+ genotype, while subclade 1a2 represented CFV isolates with an ISC*fe1*+, *tcyB*− genotype. It should be noted that four clade 1 samples were positive for only one of the PCRs detecting ISC*fe1*; two (CFV-ADRI-530 and CFV-SP27) were ISC1−, while CFV-BS-76 and CFV-90264 were ISC2−. One sample (CFVi-ADRI-513) was negative for both ISC1 and ISC2 PCR tests. In addition, CFVi98/25 typed as *tcyB*−, a feature typical of CFV, despite clustering in subclade 1a1. A small number of samples formed distinct outlier branches labeled as follows: clades 1−2 outlier (*n* = 1), clade 2 outlier (*n* = 4), clades 4−5 (*n* = 2), and clade 5 outlier (*n* = 1); all were typed as CFF.

**TABLE 2:** Cladal allocation of samples by host and correlation with genotype

Clade	Genotype^*a*^	Subspecies/biovar	Host					Total
			Bovine	Ovine	Human	Monkey	Unknown	
1a1	ISC*fe1*+, *tcyB*+	CFVi	46	1	0	0	4	51
1a2	ISC*fe1*+, *tcyB*−	CFV	34	0	0	0	5	39
1b	ISC*fe1*+, *tcyB*+	CFVi	23	0	0	0	5	28
2	ISC*fe1*−, *tcyB*+	CFF	7	1	43	0	28	79
2 outlier	ISC*fe1*−, *tcyB*+	CFF	1	0	3	0	0	4
1–2 outlier	ISC*fe1*−, *tcyB*+	CFF	0	0	1	0	0	1
3	ISC*fe1*−, *tcyB*+	CFF	2	0	7	0	7	16
4	ISC*fe1*−, *tcyB*+	CFF	6	6	9	0	26	47
4–5 outlier	ISC*fe1*−, *tcyB*+	CFF	0	1	0	0	1	2
5	ISC*fe1*−, *tcyB*+	CFF	13	0	2	0	5	20
5 outlier	ISC*fe1*−, *tcyB*+	CFF	0	0	1	0	0	1
6	ISC*fe1*−, *tcyB*+	CFF	0	0	14	0	0	14
7	ISC*fe1*−, *tcyB*+	CFF	6	10	6	1	6	29
								331

^*a*^ Determined by *in silico* PCR.

Comparison of the phenotypic classification, as generated at AHML, and phylogenetic placement of the 33 CF samples indicated congruence for 31 samples (Table 1). The 10 CFV isolates all grouped in clade 1a2, clades 1a1 and 1b included 5 CFVi isolates each, while 11 CFFs were distributed across clades 2–7. The two samples that deviated from this pattern included ADRI-1359, which clustered in clade 1a1 despite typing as CFF phenotypically, and 19AI-311, which associated with clade 7 despite biochemical typing as CFVi. These results demonstrated substantial interlaboratory differences in CF typing, as well as challenges in CF typing, even using standardized biochemical methods within a single laboratory. Indeed, when the entire CF collection was considered, numerous discrepancies were found between phenotypic and genotypic subspecies assignments (Table 3; Table S1). Most inconsistencies concerned samples phenotypically assigned to CFF, which were genotyped as CFVi (*n* = 38), while 10 samples identified as CFV were genetically typed as CFVi. All but 2 of the 91 samples not identified to subspecies in the NIH database were genotyped as CFF.

**TABLE 3:** Comparison of reported subspecies and biovar type compared to genetic identification^*a*^

Reported subspecies and biovar	Subspecies and biovar based on genetic analysis^*b*^			Total
	CFF	CFV	CFVi	
CFF	121	1	38	160
CFV	2	37	10	49
CFVi	1	0	30	31
CF	89	1	1	91
				331

^*a*^ CF, samples not identified to subtype.

^*b*^ Includes phylogenetic placement and *in silico* PCR.

### Gene screening of the *C. fetus* genome collection

BLAST screening of the entire collection of 331 genomes (Table S4) confirmed that all isolates of clades 1, 2, 3, 6, and 7 were serogroup A, except for two CFVi isolates of clade 1a1, which scored as AB, while all isolates of clades 4 and 5 were serotype B. The IS605 transposase, distinct from the ISCFe1 sequence of transposon IS607, was found to occur exclusively in all isolates of clade 1, including CFVi-ADRI-513.

Prompted by the previous identification of multiple distinct *dam* gene homologs in 14 polished mammalian-associated CF genomes (11), a comprehensive review of all the adenine MTase genes present in these 14 genomes was undertaken. Based upon screening in the REBASE database and NCBI annotation, eight such genes were identified (Table 4).

**TABLE 4:** Methyltransferase (MTase) genes^*a*^ and ORF location in 14 *C. fetus* isolates

Isolate	Genbank accession	Clade	Type I R-M subunit M/hsdM	Type I N-6 DNA methylase/cfeMII	Type II Adenine-specific MTase/cfeMI	Type II site-specifc MTase/cfeMIV	Type II DNA adenine methylase dam1 allele	Type II DNA adenine methylase/dam2a allele	Type II DNA adenine methylase/dam2b allele	Type II DNA adenine methylase/dam3 allele	Type II DNA MTase/EcoRIM	Type II R-M MTase/cjeMI	Type III site-specifc MTase/cfeMIII
CFVi-03-293	CP006999 (genome) CP007000 (plasmid)	1a1	RS03855		RS04645			A0102 (plasmid)	RS05255		RS06210 RS09090	RS07200	
CFVi-ADRI-545	CP059432	1a1	4520		5720			4735	003200^*c*^ 004035^*c*^ 005485		10,655	2120	
CFV-84-112	HG004426	1a2	10040		11630			07060 13340		6070	7550^*b*^ 19220	15,310	
CFV-97-608	CP008810	1a2	1048		892			0726^*c*^ 1340		463	1900	1531^*b*^	
CFV-NCTC10354	CP043435	1a2	984		828					465	1226 1845	1425	
CFV-08AI-948	CP059441	1a2	5630		4645			003445 003830	5060	2445	9695	7780	
CFV-08AI-1102	CP059439	1a2	5440		4655			003450 003835	6440	2445	9755	7770	
CFVi-ADRI-545	CP059437	1b	5890		004925^*b*^			000825^*c*^ 002015^*c*^ 004170		2810		8285	
CFF-82-40	CP000487	2	990		827		670					1393	
CFF-09AI-980	CP059445	2	4845	006125^*c*^	4065		3305					007040^*b*^	
CFF-00AI-031	CP059443	2	4885		4105		3345					006,840(*P*)	
CFF-NCTC10842	LS483431	4			828	326	665					01367^*b*^	
CFF-04-554	CP008808	5			RS04150	RS01605		RS05495(*P*)				RS09940(*P*)	
CFF-02AI-725	CP059974	7	4825		4025			5905				007030(*P*)	3840

^*a*^ Due to some inconsistencies in the annotation of these ORFs, some genes have been renamed. *P*, pseudogene.

^*b*^ Truncated at C terminal.

^*c*^ Based on NCBI annotation only.

**Fig 2:**
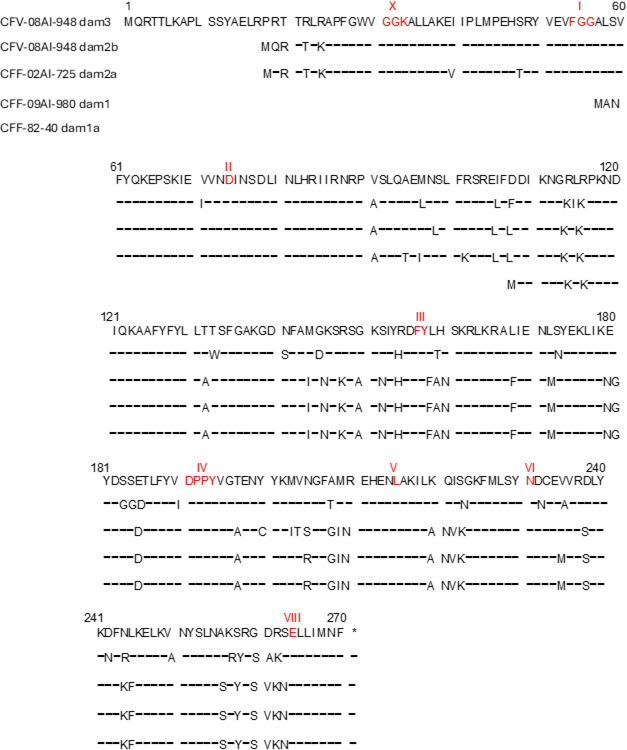
Alignment of the predicted translation products of all *dam* genes. Important motifs previously identified for methyltransferase activity (59) are highlighted in red.

One gene (*hsdM*) encoded a methylase associated with a Type I R-M enzyme, which had a homolog in *Campylobacter concisus*. Another gene (*cfeMIII*) encoded a type III site-specific methylase (SS MTase); homologs to this gene were found in *C. fetus* subsp. *testudinum* (75% identity) and *Helicobacter pylori* (61% identity). Of the five genes that encoded type II methylases, two encoded proteins with good sequence identity with other products that targeted either a GAATTC (*EcoRIM*) motif or the closely related RAATTY (c*feMI*) motif. For example, the *EcoR1M* product exhibited 87% identity to a *Campylobacter iguaniarum* homolog targeting GAATTC, while the *cfeM1* product was 67% identical to a homolog of *Campylobacter hyointestinalis* (NCTC11608) reported to recognize RAATTY. The *cjeMI* product was less conserved, having 51% identity to a *C. coli* homolog. The *cfeMIV* gene, previously annotated as *dpnA*, encodes a type II site-specific DNA methylase (TII SS MTase), which has 86% identity to a *C. hyointestinalis* homolog predicted to target the GATC motif. As described previously, the remaining gene, which is predicted to encode a DNA adenine methylase (*Dam*), appears to present as multiple alleles of differing lengths, both within and between isolates (Fig. 2). The *dam* gene of CFF-82-40 was predicted to encode a protein of 163 amino acids, but coding predictions of other samples with a similar sequence suggest the use of an upstream initiation codon, which would yield a protein of 213 amino acids; accordingly, all these sequences are assigned as *dam1*. Other genes with distinct upstream sequences and initiation codons were identified. Genes encoding a 253 amino acid protein existed as two distinct alleles, *dam*2a and *dam*2b, while a gene designated as *dam*3 encodes a 270 amino acid product. The *dam3* product was similar to the *dam*-encoded proteins in *Campylobacter mucosalis* (78% identity) and *C. coli* (65% identity), while the *dam2b* product exhibited 85% identity to a *dam* protein in *C. hyointestinalis*. An additional gene, *cfeMII,* which is predicted to encode an N-6 DNA methylase, was identified in only one sample, CFF-09AI-980. The product of this gene was 99% identical to homologs in several isolates of *C. hyointestinalis*.

BLAST screening of the 331 *C. fetus* cohort for the presence of these DNA MTase genes employed complete gene coding sequences or, in the case of the *dam* gene, sequences corresponding to the start and stop sequence of each allele, as well as internal sequences to differentiate between *dam2a* and *dam2b* versions. As summarized in Table 5 and illustrated in the phylogeny (Fig. 1), with just a few exceptions, retention of many of these genes was highly correlated with the tree’s cladal structure. An intact *cjeMI* gene was conserved in most isolates of clade 1. Still, in the other clades, it was either absent or retained in the correct reading frame in just a proportion of isolates, with the rest having a gene that was interrupted by one or more indels. Thus, in many of these isolates, the gene would encode either a modified protein or no product at all. The *cfeMIV* gene, encoding the type II SS MTase, was found only in clades 3, 4, and 5.

**TABLE 5:** Adenine methyltransferase content of *C. fetus* isolates based on clade^*a*^

Clade	*cfeMI*	*cfeMII*	*cfeMIII*	*cfeMIV*	*cjeMI*	*dam* alleles	*EcoR1M*	*hsdM*	Number of scored sequences	Exceptions
1a1	Y	N	N	N	Y	*dam2^b^*	Y	Y	51	Most appear to have both *dam2a* and *dam2b* genes, while 19 had a single allele; 7 were *EcoR1M* negative, while another 6 had indels in this gene; 2 appeared to have indels in *cjeMI*; 1 contained a *cfeMIII* gene
1a2	Y	N	N	N	Y/N	*dam3*	Y/N	Y	39	1 lacked *dam3*; 11 also scored positive for a *dam2* allele; 8 contained indels in *cjeM1* ORF; due to indels, a complete *EcoR1M* ORF could not be confirmed in 19 isolates
1b	Y	N	N	N	Y	*dam2a, dam3*	N	Y	28	Two were *dam3*−; *cjeM1* was not confirmed in one isolate; a truncated *cfeM1* was found in one isolate
2	Y	N	N	N	Y/N	*dam1*	N	Y	79	5 contained *dam2* alleles, and 1 was *dam1*−; 21 had indels in *cjeM1*; 8 contained a complete *cfeMII* ORF
2 outlier	Y	N	N	N	N	*dam1*	N	Y	4	One was dam2b+ instead of *dam1*
1–2 outlier	Y	N	N	N	N	*dam2b*	N	Y	1	
3	Y	N	N	Y	N	N	N	Y	16	All had indels in *cjeM1*; one had an interrupted *hsdM* ORF
4	Y	N	N	Y	Y/N	*dam1*	N	N	47	5 were *dam1*−, of which 2 were *dam2b*+; 20 had indels in the *cjeM1* gene
5	Y	N	N	Y	N	N	N	N	20	One isolate was *dam2a*+, while the remainder appeared to carry a *dam2* pseudogene
4–5 outlier	Y	N	N	Y	N	Y/N	N	N	2	One isolate was *dam2a*+
5 outlier	Y	N	N	Y	N	*dam2a*	N	N	1	
6	Y	N	Y	N	N	N	N	Y	14	One was *dam2a*+; one isolate had an indel in *cfeMIII*; all *cjeM1* genes contained indels
7	Y	N	Y	N	N	*dam2a*	N	Y	29	Two isolates had an indel in *cfeMIII*;
									331	Total

^*a*^ Y, gene present; N, gene absent; and Y/N, mixed genotype between isolates.

^*b*^ Mixture of *dam2a* and *dam2b* allele types.

In contrast, the *EcoR1M* gene was present only in clades 1a1 and 1a2. The *cfeMI* gene was the only one conserved throughout, while the *hsdM* gene occurred in all clades except clades 4 and 5. The *cfeMIII* gene encoding the type III SS MTase was found only in clades 6 and 7. The *dam* gene exhibited a complicated distribution. While the copy numbers of these genes could not be reliably determined by these analyses, it was established that all members of clade 1a1 contained at least one copy of a *dam2* allele, and indeed, most of these isolates contained both *dam2a* and *dam2b* genes. Clade 1a2 members were *dam*3+, with some also containing the *dam2* sequence. Clade 1b members were *dam*2a+, and all but three also contained *dam3*. Indeed, *dam3* was restricted to these two subclades. Clades 2 and 4 generally retained *dam*1 only, except for two isolates of clade 4 and the clade 2 outlier that also contained *dam*2b. Most members of clades 3, 5, and 6 appeared to lack any functional *dam* gene, either by scoring negative when screened with all *dam* gene regions (clade 3), by the presence of a *dam2* pseudogene (clade 5), or by having an interrupted *dam2* gene (clade 6). All members of clade 7 were *dam2a+*. A complete *cfeMII* gene was found in only eight samples in the entire collection, all in clade 2; many samples in clade 1 appeared to retain only portions of this ORF.

### Methylome comparisons

To explore the impact of these genetic profiles on the methylome, PacBio sequencing data available for five samples were reanalyzed to identify methylated bases. The PacBio SMRT analysis identified several methylation motifs (Table 6).

**TABLE 6:** Methylation motifs of m6A type identified from PacBio sequencing data

Isolate	Clade	Motif string	Modified A position	Number in reference genome	Number detected	Fraction^*a*^	Group tag	Mean score	Mean Ipd ratio	Mean coverage	Objective score
CFF-02A-725	7	RAATTY	3	24,750	19,806	0.800242	RAATTY	51.06	4.92	33.08	827,536.50
		CCAAK	4	3,898	3,216	0.825039	CCAAK	58.39	6.06	35.77	157,932.50
CFF-09AI-980	2	RAATTY	3	24,750	22,169	0.895717	RAATTY	234.17	5.09	180.64	4,701,500.00
		AGCNNNNNCCT	1	364	314	0.862637	AGGNNNNNGCT/AGCNNNNNCCT	283.26	7.97	190.16	77,868.55
		AGGNNNNNGCT	1	364	314	0.862637	AGGNNNNNGCT/AGCNNNNNCCT	254.04	5.75	189.46	69,834.28
CFV-08AI-948	1a2	RAATTY	3	24,750	24,741	0.999636	RAATTY	301.60	4.97	246.93	7,459,351.00
		CTANNNNNNNTGG	3	886	884	0.997743	CTANNNNNNNTGG/ CCANNNNNNNTAG	299.69	5.14	273.47	264,385.72
		CCANNNNNNNTAG	3	886	884	0.997743	CTANNNNNNNTGG/CCANNNNNNNTAG	296.34	4.94	276.06	261,435.72
		CCAHNNNNWNAAGND	3	413	189	0.457627	CCAHNNNNWNAAGND	76.42	2.09	273.81	7,146.91
CFVi-ADRI-545	1b	CTANNNNNNNTGG	3	886	860	0.970655	CCANNNNNNNTAG/CTANNNNNNNTGG	181.23	4.76	175.86	151,738.47
		CCANNNNNNNTAG	3	886	863	0.974041	CCANNNNNNNTAG/ CTANNNNNNNTGG	180.35	4.62	176.81	152,004.81
CFVi-ADRI-1362	1a1	RAATTY	3	24,750	23,481	0.948727	RAATTY	256.99	5.10	203.28	5,755,160.50
		CTANNNNNNNTGG	3	886	743	0.8386	CTANNNNNNNTGG/CCANNNNNNNTAG	70.55	2.23	229.15	38,590.43
		CCANNNNNNNTAG	3	886	731	0.825056	CTANNNNNNNTGG/CCANNNNNNNTAG	69.48	2.15	233.52	36,793.43

^*a*^ Values represent the portion of the genome corresponding to the reference genome CFV-08AI-948.

The most common modification was RAATTY, which was identified in four of the five isolates. Notably, CFVi-ADRI-545 did not exhibit this modification. This isolate was unusual in that it lacked the *EcoRM1* ORF, while the *cfeM1* ORF was modified at its C terminus. Unlike the two CFF isolates, all three CFV/CFVi isolates were methylated at the CTANNNNNNNTGG/CCANNNNNNNTAG motif. One isolate, CFF-02AI-725, was also methylated at the motif CCAAK, and *cfeMIII* is thus a potential candidate for this methylation activity, given that this gene was found only in this isolate among those interrogated by SMRT analysis. All these motifs were methylated to high levels (>80%); the slightly lower values observed for the CFF isolates are likely due to the use of the longer CFV-08AI-948 genome as a reference and reflect some bases that are not present in the CFF isolates. The CFF-09AI-980 sample also exhibited methylation at the motif AGGNNNNNGCT/AGCNNNNNCCT, and it is notable that this is the only sample of the group to retain a copy of the *cfeMII* gene. The gene responsible for conferring methylation at the additional motif CCAHNNNNWNAAGND in sample CFV-08AI-948 is currently unknown. Most notable in this analysis is the absence of a GATC motif that we expected might be targeted by many of the *dam* gene alleles.

Further methylome analysis was performed on Nanopore sequences for seven isolates, including one additional isolate from each of clades 4 and 5 (Table 7). While this analysis was unable to detect some of the methylated motifs identified by the PacBio SMRT analysis, these data further support the universal methylation of the third base of the RAATTY motif by the *cfeM1* product in all CF samples. Indeed, this analysis also identified, with a lowered prediction score, modification of this motif in CFVi-ADRI-545, suggesting that the *cfeM1* gene modification in this isolate may not render it completely inactive.

**TABLE 7:** Methylation motifs identified from Nanopore sequence data of seven *C. fetus* isolates

Isolate	Clade	Characterized motif	Motif	Predicted position	Predicted type	Prediction score	Model
CFF-02AI-725	7	AA6mATTY	AAATTY	3	6mA	53.19	Knn, nn, rf
		GA6mATTY	GAATTY	3	6mA	57.52	
CFF-00AI-031	2	AA6mATTY	AAATTY	3	6mA	51.31	Knn, nn, rf
		GA6mATTY	GAATTY	3	6mA	56.97	
CFF-10AI-182	5	RA6mATTY	RAATTY	3	6mA	52.3	Knn, nn, rf
CFF-ATCC27374	4	AA6mATTY	AAATTY	3	6mA	53.55	Knn, nn, rf
		GA6mATTY	GAATTY	3	6mA	58.68	
CFV-08AI-948	1a2	AA6mATTY	AAATTY	3	6mA	50.98	Knn, nn, rf
		GA6mATTY	GAATTY	3	6mA	55.69	
CFV-08AI-1102	1a2	RA6mATTY	RAATTY	3	6mA	52.93	Knn, nn, rf
CFVi-ADRI-545	1b	A6mAATTY	AAATTY	2	6mA	39.23	Knn, nn, rf
		GA6mATTY	GAATTY	3	6mA	19.37	

### Genomic distribution of methylation motifs

**Fig 3:**
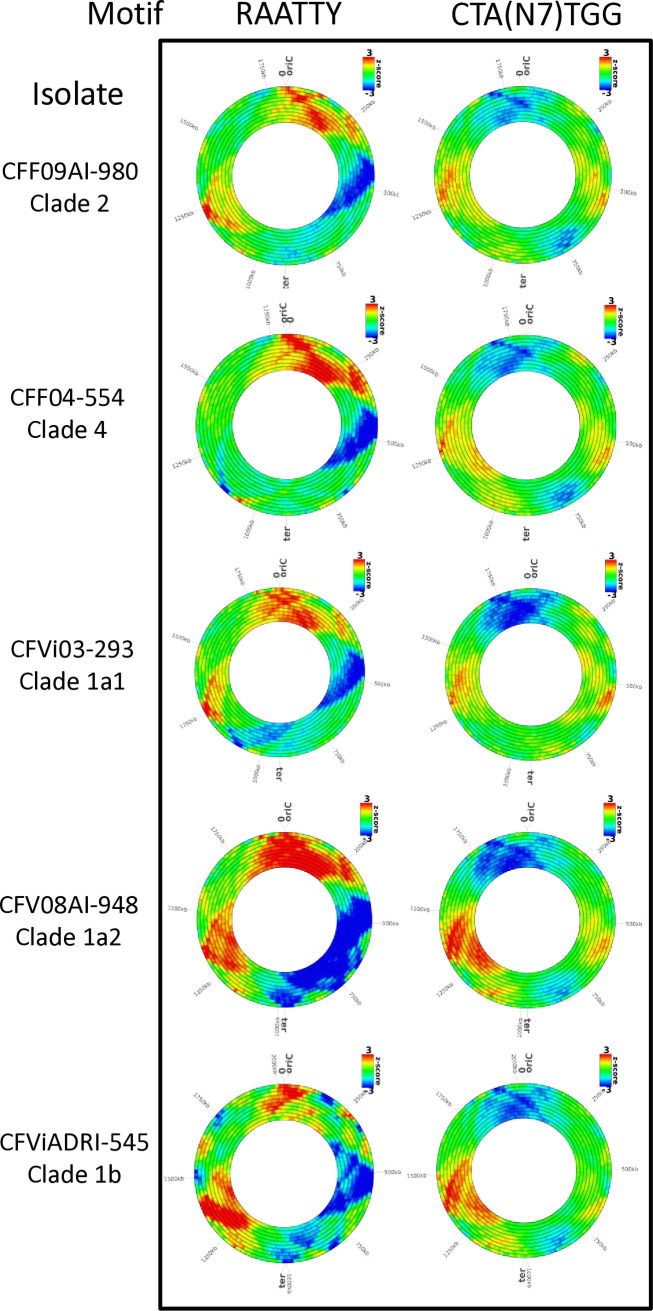
DistAMo analysis of the distribution of methylation motifs identified in CF samples. The genomes of five CF samples were analyzed for the distribution of two motifs. The different rings represent the motif distribution using window sizes ranging from 500 kb (inner ring) to 50 kb (outer ring) in 50 kb increments. Each motif distribution is color-coded according to the legend in the upper right corner; overrepresented regions (red) are scored as positive, and underrepresented regions (blue) as negative, with values of ≥2 or ≤−2 considered significant.

To explore the potential significance of these variations in adenine methylation, the genomic distribution of the sequence motifs targeted by these methylases was evaluated. Figure 3 provides a color-coded illustration of the genomic distribution of the two commonly methylated motifs for five CF members representative of several clades. These illustrations do suggest that the CFV genome contains distinct regions in which the RAATTY motif is both under- and overrepresented compared to the CFF genomes. At the same time, the results for the two CFVi isolates are less dramatic. The CFVi-03-293 isolate appears to have a region in which the CTANNNNNNNTGG motif is underrepresented compared to the other isolates.

Further review of these findings was undertaken by identifying specific genes within these genomes that scored greater than 2.0 (overrepresented) or less than −2.0 (underrepresented) for each of these motifs (Worksheets S1 to S10). In the case of the RAATTY motif, for the most part, all five CF genomes exhibited similar groups of genes in each category. Notably, the RAATTY motif was underrepresented in most of the *sapA*/*sapB* genes, in genes encoding several other membrane-associated products, such as transporters, chemotaxis proteins, and secretory systems, as well as some genes involved in various metabolic processes, including SAM-dependent methyltransferases. In addition, prophage genes, including those encoding the phage portal and tape measure proteins, were also well represented within this gene class, which was the primary reason for the underrepresentation of RAATTY in the CFV-08AI-948 genome. The lexA transcriptional repressor, which represses genes such as *recA* and *lexA* involved in the response to DNA damage, and which is present in CFV-08AI-948 and CFVi-ADRI-545, was also scored as being underrepresented by the RAATTY motif. Interestingly, the overrepresentation of this motif was found in several other DNA MTase genes (*cfeMII, cjeMI*, and *cfeMIV*), as well as in genes responsible for critical cellular processes (ribosome generation, *dnaA*) and miscellaneous metabolic functions. The relatively few genes scored as under- or overrepresented by the CTANNNNNNNTGG motif were generally similar in all five genomes, with a *lexA*-encoded family transcriptional regulator identified in the latter group in CFV-08AI-948.

## DISCUSSION

### Correlating phenotypic and genotypic types

This study has clearly shown that, even under well-controlled conditions, the traditional phenotypic methods of CF classification are challenging and can result in subtyping errors when compared to genetic typing methods. However, it is notable that phenotyping using the AHML protocol was significantly more accurate for a small cohort of 33 isolates than that performed in other laboratories. This method did not rely on growth at 42.5°C, as this was found to be inconsistent. Accordingly, the primary biochemical test to discriminate CFF and CFVi relied on the glycine tolerance test that used a cutoff of 1.1% rather than the traditional value of 1%. This change would help ensure that isolates grown at the elevated glycine concentration are truly CFF. Indeed, a high proportion of the misidentified sample sequences in the NCBI database are CFVi samples typed as CFF. The use of a 1.1% glycine cutoff may help avoid these errors in the future, where biochemical testing is the only option available.

The studies presented here agree with those of others in identifying a single clade within the CF phylogeny that represents all CFV/CFVi isolates. Furthermore, it is apparent that samples of the CFVi biovar segregate into two distinct subclades (1a1 and 1b), while CFV isolates cluster as a separate offshoot of subclade 1a (1a2). A mobile element, ISC*fe1,* also designated IS607, has been found to be specifically associated with the CFV/CFVi phenotype (15), two different versions of which are associated with clades 1a and 1b, respectively (26). In this report, we identify a second mobile element, IS605, that also associates exclusively with clade 1 members within our cohort of 331 isolates and could potentially be targeted for subspecies identification. Indeed, a single isolate, CFVi-ADRI-513, which was negative for the ISC*fe1* by two *in silico* PCR tests, was positive by BLAST testing for the IS605 transposase. Additional discrimination between CFVi and CFV isolates, which employs genetic testing for the presence/absence of the *tcyB* gene, respectively, shows excellent agreement with the cladal distribution of these isolates, with one exception; CFVi 98/25 behaves as a CFV based on its *tcy* genotype, but in other respects it types as CFVi, an inconsistency previously noted (19).

### Differences in genome methylation between phylogenetic groups

Prompted by previous analyses, the principal goal of this study was to examine the presence of genes capable of adenine methylation within this CF cohort and explore further possible differences in the methylome profile between CF subspecies. It should be acknowledged that while negative results of BLAST analysis are conclusive, searches that identify genes with one or more indels are less so, given that sequence errors could impact these results. Nevertheless, our analyses clearly suggest distinct distribution patterns in the retention of eight adenine MTase genes between the seven CF clades. Furthermore, methylome analysis of a small number of isolates revealed the use of both common and distinct methylation motifs. Our data suggest that some of these DNA MTases target specific motifs, while the target sequences for others remain unknown. We also note that, compared to other *Campylobacter* species for which methylome studies have been reported (38, 43), the number of methylation motifs identified here was small.

The only gene that was consistently retained across all clades was *cfeMI*. Comparison of the product sequence with other characterized adenine MTases strongly suggests that it methylates the A3 residue of RAATTY, a motif commonly targeted in many other *Campylobacter* species (39, 43). Indeed, methylation of the RAATTY motif was supported for all samples for which their methylome was analyzed by either Nanopore or PacBio SMRT data, except for CFVi-ADRI-545, for which this methylation was predicted only by Nanopore analysis, albeit with a lower prediction score. CFVi-ADRI-545 was the only sample in the entire CF collection that had a modified 3′-terminal sequence in the *cfeMI* ORF, and we suggest that this modification significantly reduced the enzyme’s activity. Notably, the RAATTY motif is underrepresented in prophage sequences, a feature that may have facilitated the entry and propagation of these elements, especially in clade 1 isolates that are deficient in the *cas* gene repertoire (11). A gene encoding an EcoR1M product, which likely targets the related motif GAATTC, was found in most samples of clades 1a1 and 1a2 but not in CFVi-ADRI-545 and in no other clades. It remains possible that tweaking the methylation status of this motif could have an impact on genome function; however, further investigation is needed to explore this possibility.

Another motif identified by the PacBio SMRT methylome studies included CCAAK; methylation at A4 of these sites was observed only in sample CFF-02AI-725 of clade 7. The gene encoding the Type III sequence-specific MTase (*cfeMIII*) was restricted to members of clades 6 and 7, strongly suggesting that this product was responsible for this modification. Indeed, BLAST alignment of this gene product with a portion of a ssMTase of the *H. pylori* strain hpy (hp0008), which is strongly implicated in methylation at this site (60), yielded 56% identity over a 462 amino acid window (Fig. S1).

The other methylation motif of note identified by the PacBio SMRT analysis was CTANNNNNNNTGG/CCANNNNNNNTAG, which was found in clade 1 members only. These three CFV/CFVi samples all retained *cjeMI,* while the two CFF samples studied lacked an intact ORF for this gene, thereby suggesting that the *cjeMI* product may be responsible for this modification. While virtually all clade 1 samples retain a complete *cjeMI* ORF, some CFF samples within the collection also retain this gene, although in many cases, indels appear to disrupt the reading frame.

The *dam2 and dam3* genes identified in many of these CF isolates exhibit features described for the well-characterized bacteriophage T4 and *Escherichia coli* products (59, 61), in which conserved residues in both the N- and C-terminal regions of those proteins interact to form the catalytic domain (Fig. 2). However, depending on the initiation codon employed by the *dam1* gene, its products lack the N-terminal X and I motifs and possibly also motif II. It therefore appears likely that the *dam1* product is either inactive or has a different function from that of the other *dam* genes. The pattern of the *dam* gene distribution throughout the seven clades was notable. With few exceptions, clades 2 and 4 retain *dam1* only, clade 7 consistently carries *dam2a,* while isolates of clades 3, 5, and 6 appear to lack an intact gene. In contrast, many isolates of clade 1 have multiple versions of this gene, with *dam3* found only in those of subclades 1a2 and 1b. Apart from the N-terminal truncation of *dam1*, this allele and *dam2a* share almost identical sequences, suggesting a common origin. In contrast, *dam2b* and *dam3* exhibit significant differences, thereby indicating that these alleles have been acquired separately. A clear role for these gene products was, however, not evident from the methylome analysis generated by either PacBio or Nanopore sequence interrogation. Given the cladal distribution of these genes, further evaluation of their activity is needed.

Three additional genes encoding DNA MTases were identified in this study. The *cfeMII* gene was detected only in CFF-09AI-980, and this sample was the only one found to exhibit methylation at the motif AGGNNNNNGCT/AGCNNNNNCCT, again suggesting a functional link. In contrast, the motifs targeted by *hsdM* and *cfeMIV* remain unknown.

The variety of genes present in these CF isolates and their clonal distribution support the idea that many of these genes have been acquired through horizontal gene transfer from other bacteria, a process known to be responsible for the wide distribution of methylation genes among prokaryotes (62). In particular, this may explain how the distinctive *dam* 3 allele has been obtained by organisms of clade 1, as well as the sporadic appearance of *dam2* genes in individual isolates of the other clades. Our findings also suggest that many CFF isolates have progressively lost functional *dam* genes through truncation, coding sequence interruption, or deletion, thus raising questions about the importance of this epigenetic modification in this species.

We also note that a number of these DNA MTase genes exhibit overrepresentation of motifs predicted to be targeted by other MTases, thereby raising the possibility of significant “crosstalk” in the functions of these genes and potentially resulting in complex feedback mechanisms by which the methylome profile of these organisms is controlled.

Genomic studies have, to date, failed to identify genetic factors that could explain the basis for host niche preference and pathogenesis by CF isolates. This study identifies a new avenue of investigation involving examination of the role of differential DNA methylation patterns on gene expression, which, in turn, could potentially result in distinct pathogen-host effects. Accordingly, further studies would clearly benefit from comparisons of global gene expression patterns of CFF, CFV, and CFVi samples using methods such as RNA-seq, which could help to reveal the mechanisms responsible for their phenotypic differences.

## Data Availability

The 26 *C. fetus* isolates sequenced during this study may be accessed from the NIH BioProject PRJNA604600 using the BioSample accessions: SAMN50485988–SAMN50486013. All software used in this study is detailed in Materials and Methods. Table S1 details all 331 CF samples included in these studies and their cladal assignment based on *in silico* PCR using the reagents listed in Table S2. All sequences used for BLAST searches are provided in Table S3, and the results of these analyses are shown in Table S4. Tables S6 and S7 summarize the data supporting DNA motif methylation as generated from PacBio and Nanopore sequence analysis, respectively. Worksheets S1 to S10 summarize the DistAMo results indicating under- and overrepresentation of the RAATTY and CTANNNNNNNTGG motifs, respectively, for genes in five representative CF genomes: CFF09AI980, CFF04-554, CFV08AI948, CFVi03-293, and CFViADRI545.

## SUPPLEMENTAL MATERIAL

Fig. S1 spectrum.00954-26-s0001.tif Alignment of two adenine methyltransferases.

Supplemental material spectrum.00954-26-s0002.xlsx Tables S1 to S4 and Worksheets S1 to S10.

ASM does not own the copyrights to Supplemental Material that may be linked to, or accessed through, an article. The authors have granted ASM a non-exclusive, world-wide license to publish the Supplemental Material files. Please contact the corresponding author directly for reuse.
